# Visible and Near-Infrared Spectroscopy Combined With Bayes Classifier Based on Wavelength Model Optimization Applied to Wine Multibrand Identification

**DOI:** 10.3389/fnut.2022.796463

**Published:** 2022-07-18

**Authors:** Tao Pan, Jiaqi Li, Chunli Fu, Nailiang Chang, Jiemei Chen

**Affiliations:** ^1^Department of Optoelectronic Engineering, Jinan University, Guangzhou, China; ^2^Department of Biological Engineering, Jinan University, Guangzhou, China

**Keywords:** visible and near-infrared spectroscopy, wine, multibrand identification, Bayes classifier, equidistant combination wavelength screening, wavelength step-by-step phase-out

## Abstract

The identification of high-quality wine brands can avoid adulteration and fraud and protect the rights and interests of producers and consumers. Since the main components of wine are roughly the same, the characteristic components that can distinguish wine brands are usually trace amounts and not unique. The conventional quantitative detection method for brand identification is complicated and difficult. The naive Bayes (NB) classifier is an algorithm based on probability distribution, which is simple and particularly suitable for multiclass discriminant analysis. However, the absorbance probability between spectral wavelengths is not necessarily strongly independent, which limits the application of Bayes method in spectral pattern recognition. This research proposed a Bayes classifier algorithm based on wavelength optimization. First, a large-scale wavelength screening for equidistant combination (EC) was performed, and then wavelength step-by-step phase-out (WSP) was carried out to reduce the correlation between wavelengths and improve the accuracy of Bayes discrimination. The proposed EC-WSP-Bayes method was applied to the 5-category discriminant analysis of wine brand identification based on visible and near-infrared (Vis-NIR) spectroscopy. Among them, four types of wine brands were collected from regular sales channels as identification brands. The fifth type of samples was composed of 21 other commercial brand wines and home-brewed wines from various sources, as the interference brand. The optimal EC-WSP-Bayes model was selected, the corresponding wavelength combination was 404, 600, 992, 2,070, 2,266, and 2,462 nm located in the visible light, shortwave NIR, and combination frequency regions. In modeling and independent validation, the total recognition accuracy rate (RAR_*Total*_) reached 98.1 and 97.6%, respectively. The technology is quick and easy, which is of great significance to regulate the alcohol market. The proposed model of less-wavelength and high-efficiency (*N* = 6) can provide a valuable reference for small special instruments. The proposed integrated chemometric method can reduce the correlation between wavelengths, improve the recognition accuracy, and improve the applicability of the Bayesian method.

## Introduction

High-quality wine is made through the high-quality grape variety and yeast strain after a unique process; its taste is pleasant and fragrant scent lead to extremely popular among consumers. The market demand of wine cannot be underestimated. The identification of high-quality wine brands can avoid adulteration and fraud and protect the rights and interests of producers and consumers.

Normally, the identification methods for wine brands mainly include manual identification method for wine taster and quantitative analysis method of multiple characteristic components. The manual method is obviously subjective bias and inefficient. Since the main components of wine are roughly the same, and the characteristic components that can distinguish wine brands are usually trace amounts and not unique. Therefore, the required quantitative analysis is complex and expensive.

Near-infrared (NIR) spectroscopy primarily reflects the vibration absorption of the overtones and combination frequencies of the hydrogen-containing group X-H (e.g., C–H, O–H, and N–H). This method usually does not require reagents, and it can measure samples directly, with the advantages of being quick and easy. Combined with the visible light region, visible-near infrared (Vis-NIR) spectroscopy has been applied in many fields, such as agriculture and food (1–6), environment (7, 8), and biomedicine (9–14).

The qualitative discriminant analysis of spectroscopy is based on spectral similarity of samples in the same type and spectral differences of different types of samples to perform spectral pattern recognition. For the identification of samples with small differences in component content, the qualitative discriminant analysis has more significant advantages than quantitative analysis. It has been applied in many fields, such as identification of the authenticity of rice seed (15), melon genotypes (16), transgenic sugarcane leaf (17, 18), and edible oil types (19), as well as screening for thalassemia (20) and schizophrenia (21). In recent years, Vis-NIR spectroscopy has also begun to be applied to the identification of wine (22–25), mainly focusing on the identification of wine origin. The identification of wine involves multiclass discriminant analysis of multiple spectral populations, which is more challenging than the binary classification problems, and related work is still rare. The main components of different brands of wine are roughly the same, but due to different production processes, there are still differences in the concentration ranges of some components, resulting in differences in the overall spectra of different wines. Vis-NIR spectral discriminant analysis has potential application in wine brand identification.

The partial least squares-discriminant analysis (PLS-DA) method (26), which is based on quantitative analysis of category assignment variables, is a well-performed method of binary classification discriminant analysis. When using PLS-DA to process multiclass discriminant analysis, it is necessary to perform multiple binary classification discrimination and comprehensive evaluation of errors. This process is complicated and difficult to popularize. When using principal components analysis-linear discriminant analysis (PCA-LDA) method (26) to dealing with multiclass discriminant analysis (the number of classifications is *n*), it is necessary to determine the optimal classification surface of *n*-1 dimension in the *n*-dimensional space, which is difficult and complex in mathematics. Thus, the method is difficult for the multiclass problem.

Based on probability distributions (i.e., prior, conditional, and posterior) of different spectral populations, Bayes classifier (27–32) perform spectral pattern recognition. Under certain assumptions (naive Bayes), compared with the classical multiregression-based methods (i.e., PLS-DA and PCA-LDA), this method is simpler and more suitable for multiclass discriminant analysis. Bayes method only requires calculating the prior probability that the unknown sample belongs to the *k*-th class and the conditional probability of the measured spectrum when the sample belongs to the *k*-th class. Furthermore, using the Bayes formula, the posterior probability that the measured spectrum is judged as the *k-*th sample was calculated. The key point is to use the spectral population data of each class sample to calculate the conditional probability.

The spectrum is absorbance data with multiple wavelengths. The naive Bayes (NB) method (27–31) assumes that the absorbance of each wavelength conforms to a normal distribution, and the absorbance of different wavelengths is probabilistically independent. Thus, the probability density parameter and probability multiplication can be used to calculate the abovementioned conditional probability. The calculation method is very simple. When dealing with a multiclassification problem, it is only necessary to repeatedly calculate the conditional probability of each class, and then combined with the Bayes formula, it is completed. There is no substantial dimensionality difficulty, and it is especially suitable for multiclass spectral discriminant analysis.

In the previous studies (28, 29), Vis-NIR spectroscopy combined with the NB method was used to identify unfertilized duck and chicken eggs before hatching. Moreover, a variety of spectral pretreatment methods were compared and optimized, and the prediction accuracy reached 94.54 and 91.67%, respectively. But, in some other applications, the NB method does not work well. In a previous study (32), Vis-NIR spectroscopy was applied to the detection of grapevine leafroll-associated virus 3 in a red-fruited wine grape cultivar. Both quadratic discriminant analysis (QDA) and NB methods were used for the discriminant analysis. The result of NB was significantly weaker than that of QDA.

In fact, the collinearity between adjacent wavelengths of Vis-NIR spectrum is relatively serious, and it is difficult to meet the probabilistic independence assumption of the NB method about absorbance, which affects the effect of Bayes classification. In a previous study (30), Vis-NIR spectroscopy was applied to the detection of citrus greening in citrus leaves. The classification tree, k-nearest neighbors (kNN), and NB methods were used to perform four classification discriminant analyses. The results showed that after the characteristic wavelength selection, the effect of the Bayes method was significantly improved, which was better than the other two methods. In a previous study, NIR spectroscopy combined with the NB method was applied to the identification of aflatoxin B1 in peanut. Through the screening of characteristic wavelengths, the effect of the Bayes method was significantly improved. Therefore, the use of appropriate wavelength selection can help overcome the correlation between spectral wavelengths and improve the accuracy of Bayes discrimination.

The objectives of this study were to propose a Bayes classifier algorithm based on wavelength optimization and apply the method to the 5-category discriminant analysis of wine brand with Vis-NIR spectra.

First, a large-scale wavelength screening for equidistant combination (EC) was performed (33–36), and then the wavelength step-by-step phase-out (WSP) method (14, 37) was used for secondary wavelength optimization, to reduce the dependence between wavelengths and improve the accuracy of Bayes discrimination. To get closer to the actual situation of wine brand identification in the market, the spectral discriminant models for accurately identifying four wine brands from a variety of wines were established. Among them, four types of wine brands were collected from regular sales channels as identification brands. The fifth type of samples was composed of 21 other commercial brand wines and home-brewed wines from various sources, as the interference brand.

## Experiment and Methods

### Experimental Materials, Instruments, and Measurement Methods

Four types of red wine brands were collected from regular sales channels as identification brands (not in order as I, II, III, and IV), namely, Great Wall (Hebei, China, 2018), Chile Aoyo (Lenquemira Valley, Chile, 2016), Dynasty (Tianjing, China, 2004), and Changyu (Ningxia, China, 2018) (20 bottles each, 5 samples/bottle, a total of 100 samples for each category). The grape varieties of the above four brands of red wine were all Cabernet Sauvignon. The fifth type of samples collected was regarded as the interference brands (denoted as V, 111 samples in total), which included 21 other commercial red wines of different brands and origins (one bottle each brand, 3 samples/bottle, 63 samples in total), as well as home-brewed red wines from different sources (48 bottles, 1 sample/bottle, 48 samples in total). In total, 511 samples were used for spectral measurements.

The XDS Rapid Content™ Liquid Grating Spectrometer (FOSS, Denmark) and a transmission accessory with 1 mm cuvette were used for spectral measurement. The spectral scope ranged as 400–2,498 nm with a 2 nm wavelength interval. Wavebands of 400–1,100 and 1,100–2,498 nm were used for Si and PbS detection, respectively. Each sample was measured three times, and a total of 1,533 spectra (I, II, III, and IV: 300 each, V: 333) were obtained. The experimental temperature and humidity were 25 ± 1°C and 45 ± 1%, respectively.

### Calibration-Prediction-Validation Framework and Evaluation Indicators

A sample-independent experimental design based on calibration-prediction-validation was adopted. The calibration and prediction sets were used for modeling and parameter optimization, and the selected model was validated using the independent validation samples that were excluded in the modeling, thus objective evaluation was obtained.

Each identification brand of wine (20 bottles, 100 samples, 300 spectra) was randomly divided into calibration (8 bottles, 40 samples, 120 spectra), prediction (6 bottles, 30 samples, 90 spectra), and validation (6 bottles, 30 samples, 90 spectra) sets.

The fifth type of samples (V, interference brands) were divided as follows: 21 other commercial brand wines (21 bottles, 63 samples, 189 spectra) were randomly divided into calibration (7 bottles, 21 samples, 63 spectra), prediction (7 bottles, 21 samples, 63 spectra), and validation (7 bottles, 21 samples, 63 spectra) sets; home-brewed wines (48 bottles, 48 samples, 144 spectra) were randomly divided into calibration (18 bottles, 18 samples, 54 spectra), prediction (15 bottles, 15 samples, 45 spectra), and validation (15 bottles, 15 samples, 45 spectra) sets; the total was calibration (39 samples, 117 spectra), prediction (36 samples, 108 spectra), and validation (36 samples, 108 spectra) sets. The calibration-prediction-validation division for the spectra of five types of samples was shown in Table 1.

**TABLE 1:** Calibration-prediction-validation division for the spectra of five types of samples.

	I	II	III	IV	V	Total
Calibration	120	120	120	120	117	597
Prediction	90	90	90	90	108	468
Validation	90	90	90	90	108	468
Total	300	300	300	300	333	1533

Referring to the previous studies (15, 27, 38), the evaluation indicators were set as the recognition accuracy rate of each type sample (RAR*_*i*_*, *i* = 1,2,…,5) and their standard deviation (RAR_*SD*_), as well as total recognition accuracy rate (RAR_*Total*_) of all samples, as follows:


(1) $${\text{RAR}}_{i}=\frac{\tilde{M_{i}}}{M_{i}},i=1,2,\ldots ,5$$

(2) RARTotal=Σ5i=1Mi~Σ5i=1Mi

where *M*_*i*_(*i* = 1, …, 5) was the number of samples of *i*-th category of the prediction set (or validation set), and $\tilde{M_{i}}$ was the number of accurately identified samples in *i*-th category samples of the prediction set (or validation set). In the modeling process, to consider the balance, wavelength models were preferred according to a comprehensive indicator (RAR_Total_ − RAR_SD_).

### Spectral Algorithm Framework of Bayes Classifier

Bayes classifier (27) is a well-known classification method based on probability theory, which is easy to calculate and is very suitable for multiclass discrimination problems. For multiclass spectral discriminant analysis, the calculation formula of the Bayes classifier is as follows:


(3) P(Class=k|Spectrum)=P(Sepctrum|Class=k)P(Class=k)Σ5i=1P(Spectrum|Class=i)P(Class=i), ⁢k=1,…,5

where *P* (Class = *k* | Spectrum) was the posterior probability that the measured spectrum was judged as the *k*-th sample; *P*(Class = *k*) was the prior probability that the unknown sample belongs to the *k*-th class; and *P* (Sepctrum | Class = *k*) was the conditional probability of the measured spectrum when the sample belongs to the *k*-th class. Finally, the category of the unknown sample was determined according to the maximum of posterior probability *P* (Class = *k* | Spectrum). The difficulty of this algorithm lies in the calculation of the conditional probability *P* (Sepctrum | Class = *k*) based on the spectrum because it involves the problem of high-dimensional multivariate probability distribution associated with many wavelengths.

The NB method is based on the assumption that the absorbance of a single wavelength conforms to the normal distribution, and the absorbance of different wavelengths is probabilistically independent. The method can decompose the multivariate probability distribution problem into multiple independent unary probability distribution problems, which is easy to calculate and popularize. To avoid the overflow of a large amount of data calculation, this article made appropriate improvements to the method. The specific steps are as follows:

1) Calculation of prior probability *P*(Class = *k*): according to the percentage of the total number of samples in the calibration set or assign equal probability to each type of sample.
2) Calculation of conditional probability *P*(Spectrum| Class = *k*): the corresponding wavelength model contained *s* wavelengths, denoted as λ_1_,…,λ_*s*_; according to the assumption that the absorbance at a single wavelength obeys a normal distribution, the mathematical expectation and standard deviation of the absorbance of each type of sample in calibration set were calculated at each wavelength *λ_*i*_*; furthermore, at each *λ_*i*_*, the probability density was used to calculate the conditional probability *P*(Spectrum*_*i*_*| Class = *k*) (*i* = 1, …, *s*) of the corresponding absorbance value; according to the assumption of independence, the probability multiplication was used to calculate the conditional probability, as follows:

(4) $$P(\text{Spectrum}|\text{Class}=k)=\prod_{i=1}^{s}P(\text{Spectru}{\text{m}}_{i}|\text{Class}=k)$$

Notably, to avoid calculation overflow, the following calculation was proposed:


(5) $$\text{ln }(P(\text{Spectrum}|\text{Class}=k))=\sum_{i=1}^{s}\text{ln}(P(\text{Spectru}{\text{m}}_{i}|\text{Class}=k))$$

3) Finally, the unknown sample was judged as the category corresponding to the maximum value of ln(*P*(Spectrum| Class = *k*)).

### Equidistant Combination-Bayes Method

As we know, the NB method (27–31) is based on the assumption that the absorbances of different wavelengths are probabilistically independent. However, the absorbance probability between spectral wavelengths is not necessarily strongly independent, which limits the application of Bayes method in spectral pattern recognition. In previous studies (33–36), the wavelength screening method of EC combined with multiple linear regression and PLS regression can overcome the collinearity of the spectrum and has been successfully applied to the quantitative analysis of multiple objects in the NIR spectroscopy. Drawing on the above research, the wavelength screening method of EC is integrated with the Bayes classifier algorithm (denoted as EC-Bayes) to reduce the correlation between wavelengths, improve the recognition accuracy, and reduce the complexity of the model.

The EC-Bayes method used all equidistant wavelength models in a wavelength range to establish Bayes models. The search parameters were set as follows: (1) the initial wavelength (*I*), (2) number of wavelengths (*N*), and (3) number of wavelength gaps (*G*). Then, the optimal wavelength model was selected based on the comprehensive indicator (RAR_Total_ − RAR_SD_).

In this research, the whole spectral region (400–2,498 nm) was used as the screening of the EC-Bayes method. The parameters *I*, *N*, and *G* were set as *I*∈ {400,402,…,2,498}; *N*∈{1,2,…,1,050}; and *G*∈ {1,2,…,50}. Furthermore, the ending wavelength (*E*) was determined as follows:


(6) E=I+2⁢(N-1)⁢G

### Equidistant Combination-Wavelength Step-by-Step Phase-Out-Bayes Method

Wavelength step-by-step phase-out is a well-executed secondary wavelength optimization method (14, 37). In this study, it is further used to improve the preferred EC-Bayes model (denoted as EC-WSP-Bayes). It can eliminate the interference wavelengths in the wavelength models obtained by optimization strategy of EC-Bayes. The algorithm framework is as follows: first, each time eliminated a wavelength, whose removing resulted in the best recognition accuracy, until only one wavelength remained; then, the optimal model was selected from the above-mentioned process of wavelengths elimination by step-by-step phase-out mode [refer to Ref. (14, 37) for details].

**FIGURE 1:**
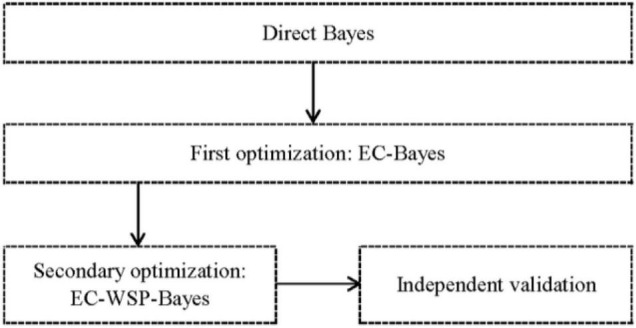
Schematic diagram of modeling framework.

The computer algorithms for the above-mentioned methods were designed using the MATLAB version 2016b software. Moreover, the schematic diagram of modeling framework is shown in Figure 1.

## Results and Discussion

### Direct Bayes Model

**FIGURE 2:**
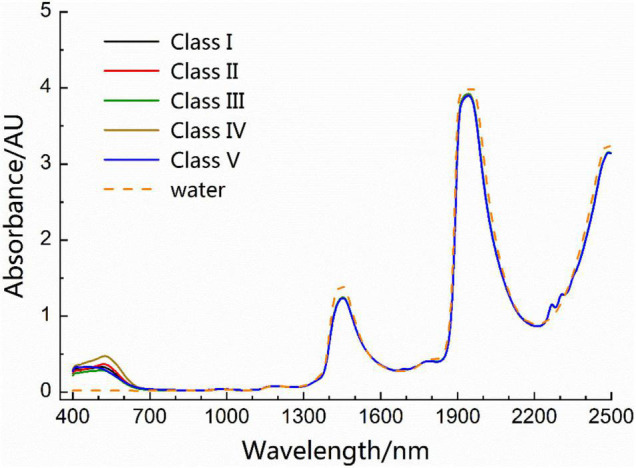
Average spectra of five types of wine and the spectrum of distilled water in the visible and near-infrared (Vis-NIR) region.

The average spectra of five types of wine in the Vis-NIR region (400–2,498 nm) are illustrated in Figure 2. In general, the composition of red wine can be broadly represented on a w/w basis as 86% water, 11% ethanol, and 3% for the remainder, which includes sugars, phenols, organic acids, and many other low-content components (22). For comparison, the spectrum of distilled water is also shown in Figure 2 (dashed line). Comparing the spectra of wine and distilled water in Figure 2, it can be observed that the two strong absorptions near 1,450 and 1,930 nm in the wine spectrum correspond to the absorption of water molecules. At 2,100–2,400 nm in the combination frequency region, and 400–700 nm in the visible light region, the weak absorption of components other than moisture was observed. The average spectrum of the five types of spectra was not significantly different in the NIR region (780–2,498 nm).

Based on the entire scanning region (400–2,498 nm, *N* = 1,050), the direct Bayes model was established first. The RAR_*Total*_ of modeling was 95.1%, and the RAR*_*i*_* of the five types were 92.2, 100.0, 94.4, 94.4, and 94.4%, respectively (refer also to Table 2).

**TABLE 2:** Recognition accuracy rates (%) of direct Bayes model in modeling.

Method	*N*	RAR_1_	RAR_2_	RAR_3_	RAR_4_	RAR_5_	RAR_*Total*_	RAR_*SD*_
Bayes	1050	92.2%	100.0%	94.4%	94.4%	94.4%	95.1%	2.9%

### Equidistant Combination-Bayes Model

The EC-Bayes method was used for wavelength model optimization based on the selection of multiparameter combination (*I*, *N*, and *G*). The parameters of the optimal model were *I* = 404 nm, *N* = 22, and *G* = 49. According to Eq. 6, the corresponding ending wavelength *E* was 2,462 nm. The RAR*_*i*_* of the five types were 93.3, 100.0, 95.6, 95.6, and 100.0%, respectively, and the RAR_*Total*_ of modeling increased to 97.0% (refer also to Table 3).

**TABLE 3:** Recognition accuracy rates (%) of the optimal equidistant combination (EC)-Bayes model in modeling.

Method	*I*	*E*	*N*	*G*	RAR_1_	RAR_2_	RAR_3_	RAR_4_	RAR_5_	RAR_*Total*_	RAR_*SD*_
EC-Bayes	404	2462	22	49	93.3%	100.0%	95.6%	95.6%	100.0%	97.0%	2.7%

The number of wavelengths (*N* = 22) of the optimal EC-Bayes model was only 2.1% of the direct Bayes model (*N* = 1,050). The wavelength model was greatly simplified, but the discrimination effect was improved.

### Equidistant Combination-Wavelength Step-by-Step Phase-Out-Bayes Model

Since the models processed by the EC-Bayes method were likely to still contain redundant wavelengths, the EC-WSP-Bayes method discussed in Section “Equidistant Combination-Wavelength Step-by-Step Phase-Out-Bayes Method” was further used to improve the selected EC-Bayes models. Specifically, the Top 10 EC-Bayes models were selected according to a comprehensive indicator (RAR_Total_ − RAR_SD_) in order from largest to smallest. Then, the corresponding 10 optimized EC-WSP-Bayes models were determined; furthermore, the optimal EC-WSP-Bayes model was determined from them.

**FIGURE 3:**
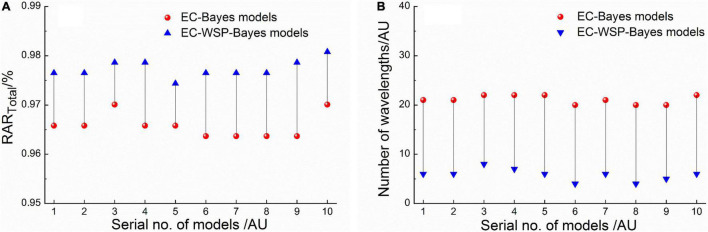
Comparison of the top 10 equidistant combination (EC)-Bayes models and corresponding EC-wavelength step-by-step phase-out (WSP)-Bayes models: **(A)** RAR_*Total*_; **(B)** number of wavelengths.

The modeling effects (RAR_*Total*_) and the number of wavelengths of the Top10 EC-Bayes models and corresponding EC-WSP-Bayes models are shown in Figure 3. It illustrated that for all of the Top 10 EC-Bayes models, after the process of WSP, the number of wavelengths was all greatly reduced, and the discrimination effects were all improved.

**FIGURE 4:**
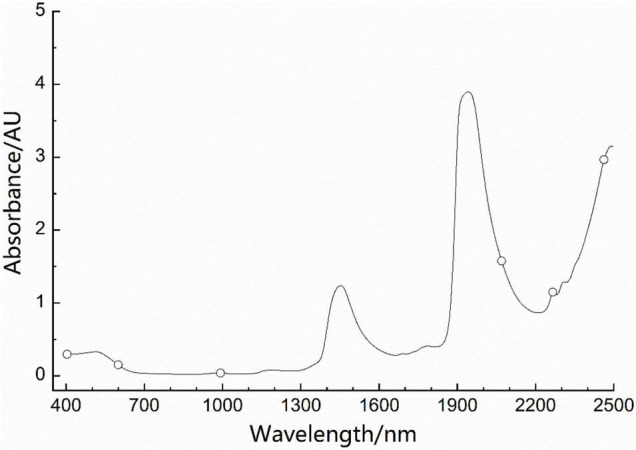
Position of the wavelength combination of the optimal equidistant combination (EC)-wavelength step-by-step phase-out (WSP)-Bayes model labeled in the average spectrum.

**FIGURE 5:**
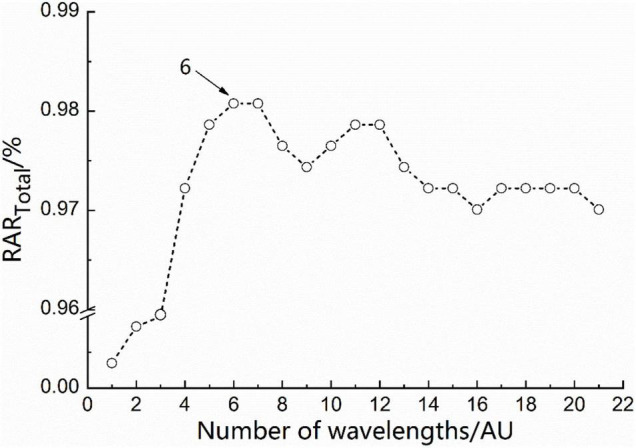
Total recognition accuracy rate (RAR_*Total*_) in the process of wavelength step-by-step phase-out (WSP) for the equidistant combination (EC)-Bayes model.

The optimal EC-WSP-Bayes model was selected (No. 10, *N* = 6), the corresponding wavelength combination was 404, 600, 992, 2,070, 2,266, and 2,462 nm located in the visible light, shortwave NIR, and combination frequency regions. The position of the wavelength combination of the optimal EC-WSP-Bayes model labeled in the average spectrum is showed in Figure 4. The RAR*_*i*_* of the five types were 94.4, 100.0, 100.0, 95.6, and 100.0%, respectively, and the RAR_*Total*_ of modeling further increased to 98.1% (refer also to Table 4). Figure 5 shows the values of RAR_*Total*_ in the process of WSP for the EC-Bayes model (No. 10), which reached maximum when *N* = 6.

**TABLE 4:** Recognition accuracy rates (%) of the optimal equidistant combination (EC)-wavelength step-by-step phase-out (WSP)-Bayes model in modeling.

Method	*I*	*E*	*N*	RAR_1_	RAR_2_	RAR_3_	RAR_4_	RAR_5_	RAR_*Total*_	RAR_*SD*_
EC-WSP-Bayes	404	2462	6	94.4%	100.0%	100.0%	95.6%	100.0%	98.1%	2.8%

Notably, the wavelength combination of the optimal EC-WSP-Bayes model is greatly simple and effective, which indicated that the WSP method is very necessary. The corresponding wavelength combination has reference value for the development of small special instrument.

### Independent Validation

A total of 468 spectra of the validation samples (90 for each of I, II, III, and IV, 108 for V), who did not participate in the modeling process, were used to validate the effect of the optimal EC-WSP-Bayes model. Using the mathematical expectation and standard deviation of the spectral absorbance in the calibration set, the conditional probability of the spectra in validation set was calculated, and the type of validation samples was determined. In validation, the RAR*_*i*_* of the five types were 93.3, 100.0, 97.8, 100.0, and 97.2%, respectively, and the RAR_*Total*_ was 97.6% (refer also to Table 5). The results showed that the optimal EC-WSP-Bayes model also achieved a good performance in validation.

**TABLE 5:** Recognition accuracy rates (%) of optimal equidistant combination (EC)-wavelength step-by-step phase-out (WSP)-Bayes model in validation.

Method	*I*	*E*	*N*	RAR_1_	RAR_2_	RAR_3_	RAR_4_	RAR_5_	RAR_*Total*_	RAR_*SD*_
EC-WSP-Bayes	404	2462	6	93.3%	100.0%	97.8%	100.0%	97.2%	97.6%	2.7%

**FIGURE 6:**
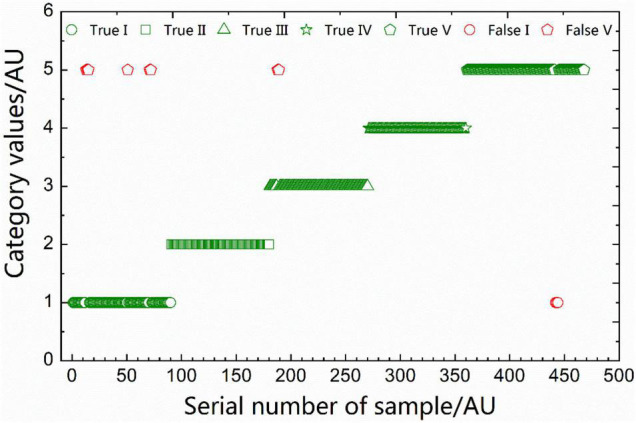
Identification for the spectra of the validation samples based on the optimal equidistant combination (EC)-wavelength step-by-step phase-out (WSP)-Bayes model.

To facilitate the observation of the identification of the validation sample’s spectra, the class *i* samples were assigned the categorical value *i*, *i* = 1, 2, …, 5, respectively. Using the optimal EC-WSP-Bayes model, the correctness of the identification for the spectra of the validation samples is shown in Figure 6. Among them, 6 spectra of type 1 samples and 2 spectra of type 3 samples were misjudged as type 5; 3 spectra of type 5 samples were misjudged as type 1; and the remaining 457 spectra were all correctly identified.

It is worth mentioning that, to get closer to the actual situation of wine brand identification in the market, this study adopted the following experimental design: four types of wine brands were collected from regular sales channels as identification brands. The fifth type of samples was composed of 21 other commercial brand wines and home-brewed wines from various sources, as the interference brand. As more brands of wine are involved, the 5-category problem in this study is more difficult than the 5-category problem for pure samples, but it is closer to the actual situation.

The article proposed an integrated optimization method (EC-Bayes, EC-WSP-Bayes), which improved the existing NB method and can overcome the independence requirement of the NB algorithm framework. The aim in this study was to compare the discriminative performance of the existing NB and the improved method. Furthermore, WSP was a simple backward elimination method. EC-WSP-Bayes made the secondary optimization for the Top 10 models of EC-Bayes by using WSP. Through the independent validation of the 5-category models of wine brands, the EC-WSP-Bayes method achieved significantly better discriminant effect, and the wavelength model was more concise. This method can be applied to wider fields in food and nutrition.

## Conclusion

The NB classifier is an algorithm based on probability distribution, which is simple and particularly suitable for multiclass discriminant analysis. However, the absorbance probability between spectral wavelengths is not necessarily strongly independent, which limits the application of the Bayes method in spectral pattern recognition.

In this study, a Bayes classifier algorithm based on wavelength optimization was proposed and applied to the 5-category discriminant analysis of wine brand with Vis-NIR spectra. The Bayes classifier algorithm was integrated with the wavelength screening methods of EC and WSP, which reduced the correlation between wavelengths, improved the recognition accuracy, and improved the applicability of the Bayesian method. In the 5-category discriminant analysis of wine brands, the total discrimination accuracy of the validation set reached 97.6%. The proposed model of less-wavelength (*N* = 6) and high-efficiency provided a valuable reference for small special instruments. The proposed Bayes classifier algorithm with wavelength optimization is simpler and efficient compared with the classical Bayes method and is also expected to be applied to spectral discriminant analysis in other fields.

The technology is quick and easy and has potential in food characterization, traceability, and authenticity of food matrices, such as protected geographical indication (PGI) and protected designation of origin (PDO) of food products, which is of great significance to food safety and nutrition.

## Data Availability Statement

The original contributions presented in this study are included in the article/supplementary material, further inquiries can be directed to the corresponding author.

## Author Contributions

TP and JC contributed to conception and design of the study. JL, CF, and NC organized the database. JL, CF, NC, and TP performed the statistical analysis. TP and JL wrote the first draft of the manuscript. All authors wrote sections of the manuscript, contributed to manuscript revision, read, and approved the submitted version.

## Conflict of Interest

The authors declare that the research was conducted in the absence of any commercial or financial relationships that could be construed as a potential conflict of interest.

## Publisher’s Note

All claims expressed in this article are solely those of the authors and do not necessarily represent those of their affiliated organizations, or those of the publisher, the editors and the reviewers. Any product that may be evaluated in this article, or claim that may be made by its manufacturer, is not guaranteed or endorsed by the publisher.
